# Electronic Health Record–Based Phenotyping for Obsessive-Compulsive Disorder: Algorithm Development and Multicenter Validation Study

**DOI:** 10.2196/89213

**Published:** 2026-10-02

**Authors:** Bo Wang, Tyne W Miller-Fleming, Dongmei Yu, Donald Hucks, Emily Gantz, Rebecca Johnston, Angela Maxwell-Horn, Nancy Cox, James Sutcliffe, Carol A Mathews, Evonne McArthur, Patrick L McGuire, Dia Kabir, Ashley Dankese, Evan J Giangrande, Rebecca G Fortgang, Shirley B Wang, Rakesh Karmacharya, Joshua L Roffman, Jeremiah M Scharf, Jordan W Smoller, Takahiro Soda, James J Crowley, Lea K Davis

**Affiliations:** 1 Massachusetts General Hospital, Richard B. Simches Research Building, 185 Cambridge Street, 2nd Floor, Boston, MA, 02141, United States; 2 Broad Institute of MIT and Harvard, Boston, MA, United States; 3 Department of Psychiatry, Harvard Medical School, Boston, MA, United States; 4 Vanderbilt Genetics Institute, Vanderbilt University, Nashville, TN, United States; 5 Vanderbilt University Medical Center, Nashville, TN, United States; 6 Department of Psychiatry, University of Florida, Gainesville, FL, United States; 7 Brigham and Women's Faulkner Hospital, Boston, MA, United States; 8 Department of Psychology, Yale University, New Haven, CT, United States; 9 University of Florida Genetics Institute, Gainesville, FL, United States; 10 Center for Autism and Neurodevelopment, University of Florida, , Gainesvilles, FL, United States; 11 Department of Genetics, University of North Carolina at Chapel Hill, Chapel Hill, NC, United States; 12 Department of Medicine, Icahn School of Medicine at Mount Sinai, 3 E 101st St, New York, NY, 10029, United States, 1 2122416500; 13 New York Genome Center, New York, NY, United States

**Keywords:** obsessive-compulsive disorder, electronic health records, natural language processing, EHR phenotyping, psychiatric genetics, polygenic scores

## Abstract

**Background:**

Obsessive-compulsive disorder (OCD) is a common psychiatric disorder, with two-thirds of affected individuals reporting severe impairment. Despite its substantial burden and moderate heritability, the etiology of OCD remains poorly understood, and treatments are often suboptimal. Although recent genome-wide association studies (GWAS) have identified some risk loci, much of the genetic architecture of OCD remains undiscovered, underscoring the need for scalable approaches to identify large, well-defined patient cohorts.

**Objective:**

This study aimed to develop and validate a scalable electronic health record (EHR)–based phenotyping algorithm for identifying OCD cases to support large-scale genetic and translational research.

**Methods:**

We leveraged EHR-linked biobank data from 2 large hospital systems, namely Vanderbilt University Medical Center (VUMC) and Mass General Brigham (MGB), to develop a high-throughput phenotyping algorithm integrating diagnostic codes, medication records, and natural language processing (NLP) of clinical notes. Algorithm performance was evaluated through expert chart review, and genetic analyses were performed in individuals of European genetic ancestry using the polygenic scores (PGS) of OCD, major depressive disorder (MDD), and height derived from the most recent GWAS.

**Results:**

Expert chart reviews demonstrated our algorithm combining both *International Statistical Classification of Diseases* (*ICD*) codes and NLP achieved the highest positive predictive values (PPV) for OCD case identification (0.84 at VUMC; 0.91 at MGB) compared to using either *ICD* codes or NLP alone, albeit with reduced case yield. At both sites, algorithm-defined OCD cases of European genetic ancestry showed significantly higher OCD PGS than controls. In sensitivity analyses adjusting for MDD status, OCD PGS associations were more robust than MDD PGS associations, while height PGS showed no association, supporting the genetic plausibility and relative specificity of the phenotype.

**Conclusions:**

This study presents a scalable and cost-efficient EHR-based approach for identifying OCD cases across health systems. The algorithm achieves high PPV, and among individuals of European genetic ancestry, algorithm-defined cases show significant OCD PGS enrichment, supporting its utility for large-scale genetic studies and advancing understanding of the disorder’s complex etiology.

## Introduction

Obsessive-compulsive disorder (OCD) is a common psychiatric disorder with a lifetime prevalence of 2.3% and an annual prevalence of 1.2% [1]. It is characterized by intrusive thoughts or impulses that typically cause anxiety or distress (obsessions), and repetitive mental acts or behaviors that one feels compelled to do (compulsions). OCD is highly debilitating; two-thirds of those affected report severe impairment, averaging 45 days in the past year unable to carry out usual work, home, or social activities [1]. Globally, OCD was ranked the 10th most disabling illness in 1990, as measured by years lived with disability, just behind schizophrenia, owing to its high prevalence, chronic course, and pervasive symptoms [2]. Symptoms typically emerge early in life, with a mean onset at age 19 [1]. For a majority of individuals, OCD is a chronic condition, with remission achieved in only about one-fifth of cases [3]. Factors associated with lower remission rates include greater symptom severity [3,4], earlier age of onset [4], and longer illness duration [5].

Despite its significant burden, the etiology of OCD remains poorly understood, and existing treatments are suboptimal. Twin studies have consistently demonstrated higher concordance rates for OCD in monozygotic compared to dizygotic twins [6-10], with estimated heritability around 40%‐50% [9,11]. Early genome-wide association studies (GWAS) for OCD were limited by small sample sizes (<3500 cases) [12,13] but established OCD as a complex genetic trait, indicating that larger sample sizes could yield significant loci. The most recent OCD GWAS identified 30 genome-wide significant loci [14]; however, despite recent growth in sample size, OCD GWAS still include fewer cases than GWAS of several other psychiatric disorders.

National disease registers, such as those in Nordic countries, have proven effective for large-scale sample collection. Diagnostic codes within these registers have generally shown high positive predictive values (PPV; the proportion of algorithm-defined cases confirmed as true cases), around 90%. For instance, 2 register-based studies in Sweden and Denmark reported PPVs ranging from 85%‐96% [15,16]. Electronic health records (EHRs) provide an alternative for case ascertainment that is scalable and cost-efficient. Unlike clinic-based recruitment, EHR-based approaches do not require prospective patient contact, and unlike national registers, which are largely limited to Nordic countries, EHRs are widely available across health care systems and offer access to rich clinical data beyond diagnostic codes, including medication records and clinical notes. EHR-based approaches have been successfully used in psychiatric research for bipolar disorder [17], treatment-resistant depression [18], binge eating disorder [19], and autism spectrum disorder [20,21].

Existing OCD GWAS [12-14,22] have used both family and unrelated sample designs. Members of this study team recently contributed EHR-linked biobank data to a large-scale GWAS meta-analysis including 28 OCD case-control cohorts [14]. Case ascertainment strategies varied across cohorts, introducing heterogeneity. Motivated by the need to improve the quality of data contributing to future OCD GWAS, as well as other downstream applications, we sought to develop an externally validated phenotyping approach that integrates structured and unstructured EHR data, an area that remains relatively underexplored in OCD research.

In this study, we report the development and validation of a high-throughput phenotyping algorithm that integrates natural language processing (NLP), *International Statistical Classification of Diseases* (*ICD*) codes, and medication administration records to systematically identify relevant evidence and determine patient OCD status across 2 academic medical centers: Vanderbilt University Medical Center (VUMC) and Mass General Brigham (MGB). Both institutions are part of the Psychiatric Electronic Medical Record and Genomics Network (PsycheMERGE), a network of EHR-linked biobanks for precision psychiatry research. Our algorithm was rigorously validated by expert chart review, demonstrating high PPV at both centers. Applying this method, we identified 417‐1057 samples at VUMC and 335‐1379 samples at MGB, depending on the algorithmic definition. Among identified cases of European genetic ancestry with available genotype data, we observed significantly elevated polygenic scores (PGSs) derived from the latest OCD GWAS [14]. Our results demonstrate that automated EHR-based phenotyping can effectively identify OCD cases and controls with precision, and that among individuals of European genetic ancestry, the identified cases show PGS associations consistent with samples ascertained through traditional, resource-intensive methods. This approach represents a valuable tool for accelerating psychiatric genetic research.

## Methods

### Study Participants

This research was conducted as part of the Psychiatric Genomics Consortium’s (PGC) ongoing effort to identify and collect DNA samples from patients with OCD, using 2 EHR-linked biobanks within the PsycheMERGE Network: Vanderbilt University Medical Center’s biobank (BioVU) at VUMC in Nashville, Tennessee, and the MGB Biobank (MGBB) [23] at MGB in Boston, Massachusetts. The study included 213,275 individuals receiving care at VUMC from 1989 to 2021, and 97,275 patients at MGB from 1976 to 2021. Additional data details are provided in Multimedia Appendix 1.

### Ethical Considerations

Ethical approvals were obtained from the institutional review boards at both VUMC and MGB, including an informed consent waiver for the use of retrospective medical record data without patient interaction. All participants provided written informed consent for their inclusion in the respective biobanks for broad-based research use.

### OCD Phenotyping Algorithm

#### Algorithm Overview

Our OCD algorithm was developed at VUMC through an iterative, consensus-driven process, involving domain experts from PGC, VUMC psychiatry, and neurology. Structured data elements of the algorithm included *International Classification of Diseases, 9th Revision* (*ICD-9*) and *International Statistical Classification of Diseases, 10th Revision* (*ICD-10*) inclusion and exclusion codes (Tables S3-S4 in Multimedia Appendix 2), while diagnostic and treatment-related keywords were incorporated into the NLP arm (Table S5 in Multimedia Appendix 2). These keywords were identified through iterative consultation with psychiatry domain experts. To establish a broad performance base, we included *ICD* codes for body dysmorphic disorder (BDD) and trichotillomania, with the understanding that these criteria can be modified to adjust the algorithm’s specificity for different use cases. Medications were extracted from multiple sources, including structured EHR databases containing recorded prescriptions and unstructured clinical notes. Once all algorithm components were finalized and the logical order of its operations was confirmed by the domain experts, we implemented the algorithm within the VUMC clinical database. Overall, 3 rounds of chart review were conducted at VUMC and MGB, respectively, to validate its performance. Detailed steps in developing the algorithm are provided in Multimedia Appendix 1.

The resulting algorithm integrates diagnostic, medication, and clinical note data to determine patients’ OCD status. It begins with exclusion criteria (Figure 1A), followed by 2 inclusion pathways: (1) an *ICD* arm identifying relevant patients with at least one relevant *ICD-9* and *ICD-10* code for OCD or obsessive-compulsive (OC) spectrum disorders (Figure 1B); and (2) an NLP arm detecting OCD-related mentions in patients’ clinical notes (Figure 1C). Exclusion criteria include *ICD* codes for “Other specified metabolic disorders” and “Metabolic disorder, unspecified” to prevent rare but highly penetrant metabolic disorders, such as Lesch-Nyhan syndrome, from being misidentified as OCD. We derived 4 definitions of OCD from this algorithm: (1) *ICD*-defined, including cases identified exclusively through the *ICD* arm of the algorithm; (2) NLP-only, encompassing cases that lacked *ICD*-coded information but are identified through the NLP arm based on clinical notes; (3) *ICD*-or-NLP, identifying cases from either the *ICD* or the NLP arm, ie, the disjunction of the 2 arms resulting in the union of cases; and (4) *ICD*-and-NLP, requiring concordance between the 2 arms, ie, the conjunction of both arms representing the intersection of cases. We used the *ICD*-or-NLP definition to sample OCD cases for chart reviews, as it represents the broadest and most inclusive version of our algorithm.

**Figure 1.**
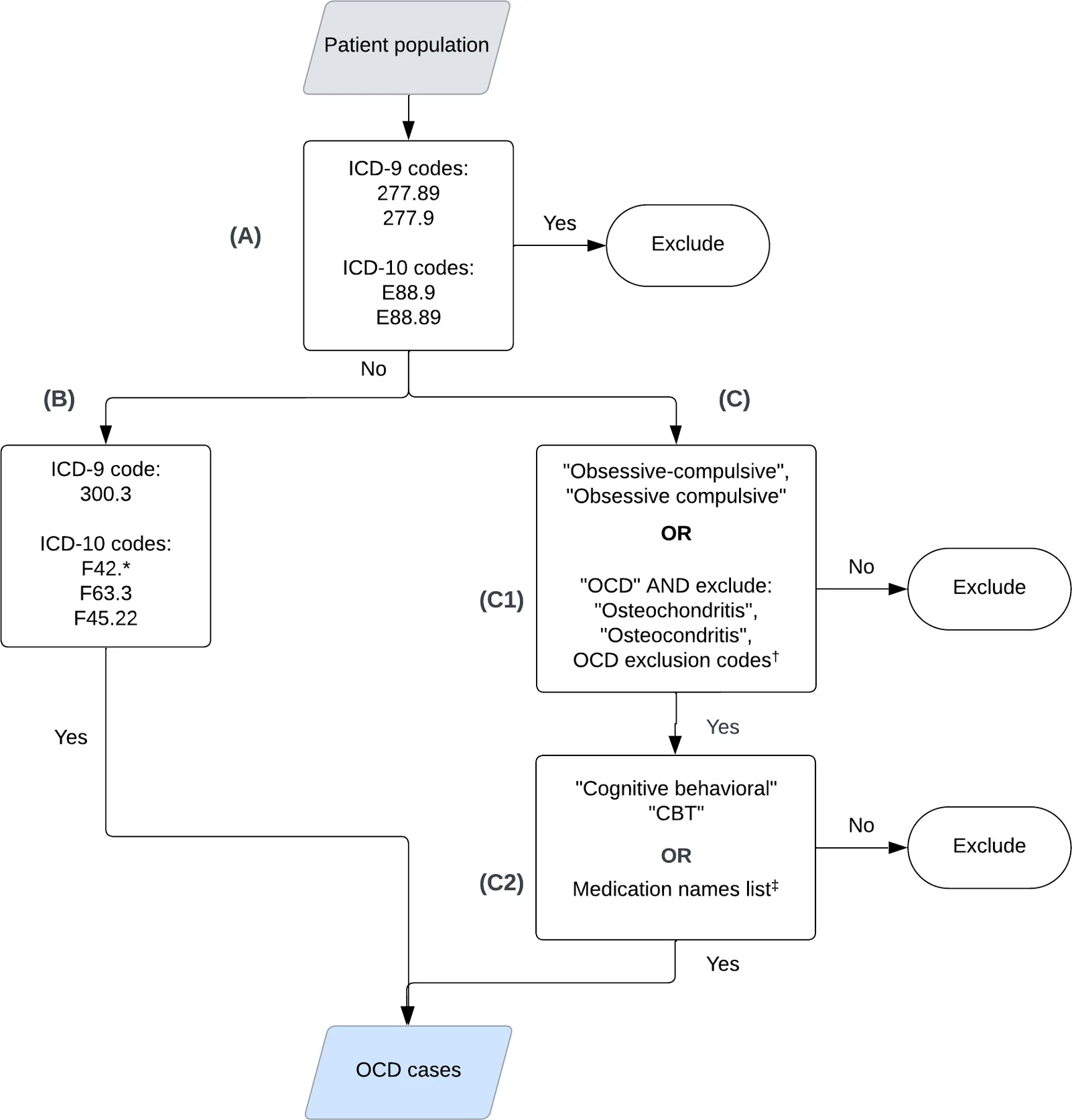
Obsessive-compulsive disorder (OCD) phenotyping algorithm flowchart at Vanderbilt University Medical Center (VUMC) and Mass General Brigham (MGB), consisting of an *International Classification of Diseases* (*ICD)* code-based exclusion criteria (A), an *ICD* code-based inclusion arm (B), and a rule-based natural language processing (NLP) algorithm arm (C). *: All *International Classification of Diseases, 10th Revision* (*ICD*-10) subcodes under F42 (ie, F42.*). †: *ICD* codes used for exclusion in the NLP arm: Table S4 in Multimedia Appendix 2. ‡: Medication names list: Table S5 in Multimedia Appendix 2. *ICD*: International Statistical Classification of Disease and Related Health Problems, OCD: obsessive-compulsive disorder.

#### NLP System

We developed a rule-based NLP system to identify OCD mentions from clinical notes (Figure 1C), comprising 2 steps: (1) detecting OCD keyword mentions (C1); and (2) identifying cognitive behavioral therapy (CBT) mentions and relevant medications (C2). To meet the criteria in C1, a patient must have at least 2 mentions of OCD on separate days in clinical documents, such as problem lists, discharge summaries, clinical communications, progress notes, or visit notes, with the first OCD mention before age 55 [24,25]. This requirement accounts for the possibility that late-life OCD diagnosis may more likely reflect changes due to cognitive decline, often with a biological etiology distinct from that of idiopathic OCD. We incorporated exclusion criteria in C1, using keywords and codes, to disambiguate the acronym “OCD” and prevent false identifications, such as mentions related to osteochondritis dissecans. In C2, mentions of OCD treatments, including CBT or relevant medications, provide additional evidence for OCD. Medications (see Multimedia Appendix 2 Table S5 for the list of medication names used) were extracted from 2 sources: (1) structured patient medication database; and (2) unstructured clinical notes using the MedEx-UIMA (The University of Texas Health Science Center at Houston and Vanderbilt University) system [26]. Further details about the NLP system are provided in Appendix S3 and Figure S1 in Multimedia Appendix 1.

### Chart Review

Two reviewers with clinical training in psychiatric assessment (EM: a postpsychiatry clerkship medical student; PM: a psychiatry resident) were selected to conduct chart reviews at VUMC. Both were trained using chart review instructions (Appendix S4 Multimedia Appendix 1), which included *Diagnostic and Statistical Manual of Mental Disorders* (Fifth Edition; *DSM-5*) diagnostic criteria for OCD, evidence grading guidelines, and defined inclusion and exclusion rules. OCD cases for chart review were identified using the *ICD*-or-NLP definition. Both reviewers independently examined charts from the same set of 25 subjects to assess interrater reliability (IRR). Reviewers, blinded to each other’s assessments, determined OCD presence and graded supporting evidence. A psychiatrist (JB) and a psychiatric nurse practitioner (HH), both with clinical expertise in OCD, adjudicated discrepancies and provided feedback. Once adequate agreement (IRR>90%) was achieved, each reviewer independently assessed an additional 25 subjects. If agreement was inadequate, another 25 subjects were reviewed, followed by the same confirmation process until the required agreement threshold was met. Once confirmed, the remaining subjects were reviewed independently.

For external validation at MGB, 2 board-certified psychiatrists (JR and RK) with extensive experience in psychiatric chart review conducted 2 review rounds for algorithm refinement and evaluation. Disagreements were adjudicated by a third experienced psychiatrist (JS). The first 2 rounds together included 68 algorithm-identified cases and 8 noncases. In the final round, 3 clinical psychologists (EG, RF, and SW) reviewed 90 randomly sampled algorithm-identified cases. All 3 reviewers first independently reviewed the same 10 charts for calibration, followed by a meeting to align on diagnostic criteria and evidence grading. Each reviewer then independently assessed 33‐35 charts, with each chart reviewed by 2 reviewers and discrepancies resolved by the third. Reviewers at both sites followed standardized chart review instructions (Appendix S4 and Figure S2 in Multimedia Appendix 1).

### Genetic Analysis (VUMC)

For genetic analysis, we used BioVU, VUMC’s biobank that links deidentified DNA samples with EHRs. BioVU, established in 2007, obtains patient consent through forms provided in outpatient clinic settings. We applied quality control measures (described in Appendix S5.1 Multimedia Appendix 1) to the algorithm-defined OCD cases and controls in BioVU to generate the final dataset used for genetic analysis. OCD cases were matched to controls at a 1:3 case-control ratio based on median age in the medical record, sex, and the first 2 genetic principal components.

The OCD PGS was derived from the OCD GWAS by Strom et al [14] (excluding 23andMe and BioVU samples). The PGS for height and major depressive disorder (MDD) were derived using GWAS summary statistics for height [27] and MDD [28], respectively. We used Polygenic Risk Score via Continuous Shrinkage (PRS-CS) [29] to estimate posterior single-nucleotide polymorphism (SNP) effect sizes, using CEU (refers to Utah residents with Northern and Western European ancestry) samples from the 1000 Genomes Project Phase 3 as the reference panel. PGSs were calculated as the sum of the risk allele dosages weighted by the posterior SNP effect sizes. Logistic regression assessed the association of the OCD, MDD, and height PGSs with algorithm-defined OCD status, adjusting for current age, genotype batch, and principal component analysis components 1‐10. Nagelkerke *R*^2^ was calculated for the full model and for the corresponding model excluding the PGS term, and the difference was reported to quantify the incremental contribution of each PGS to OCD case status. Additionally, we performed a sensitivity analysis that included MDD status as a covariate to assess whether associations between the OCD, MDD, and height PGSs and OCD case status were independent of comorbid MDD. MDD status was extracted from the EHR using a previously established algorithm [30,31], as detailed in Multimedia Appendix 1.

### Genetic Analysis (MGB)

The MGBB, established in 2010, collects biological samples, health information, and genetic data to support biomedical research [23]. Participants are enrolled using a broad-based consent process by research coordinators at clinical and public hospital locations or electronically through the MGB patient portal. Genotyping was performed for 53,853 participants in 13 batches using 4 Illumina array platforms: Multi-Ethnic Genotyping Array (MEGA, batch 1), Expanded Multi-Ethnic Genotyping Array (MEGAEX, batch 2), Multi-Ethnic Global (MEG, batches 3‐9), and Global Screening Array (GSA, batches 10‐13). Batches 8 and 9 were excluded due to few OCD cases (see Table S1 in Multimedia Appendix 1 for case and control counts). Quality control procedures are detailed in Appendix S5.2 and Figure S4 in Multimedia Appendix 1.

Consistent with methods used at VUMC, SNP effect sizes from GWAS of OCD, MDD, and height were used to construct PGSs. Posterior SNP effect sizes were calculated using PRS-CS [29], with the CEU reference panel. PGSs were calculated as weighted sums of risk allele dosages based on the posterior SNP effect sizes. Logistic regression analyses were performed separately in the MEG and GSA datasets to evaluate associations between each PGS and algorithm-defined OCD case status, adjusted for age and genetic principal components. The incremental contribution of each PGS to OCD status prediction was assessed by calculating the difference in Nagelkerke *R*^2^ between the full model and the corresponding model excluding the PGS term. Sensitivity analyses were conducted by including MDD status as a covariate to assess whether the associations of the OCD, MDD, and height PGSs with OCD case status were independent of comorbid MDD.

## Results

### Chart Review Validation

At VUMC, reviewers achieved 91% raw agreement and Cohen κ=0.72 in the first 2 rounds (50 charts total), indicating substantial IRR [32]. In the third and final round, each reviewer independently assessed 22‐25 additional charts, totaling 97 reviews. At MGB, 2 initial rounds of chart review identified 34 OCD cases and 42 noncases, with reviewers agreeing on 71 out of 76 charts (93% agreement; κ=0.91). In the final round, 3 reviewers (EG, RF, and SW) assessed 90 charts (52 cases and 38 noncases), achieving 87% agreement and κ=0.71. At MGB, only the final round was used for algorithm validation (Table 1).

**Table 1.** Comparison of the 4 obsessive-compulsive disorder (OCD) definitions in terms of positive predictive values (PPV) and the number of genotyped OCD cases identified in the biobanks.

Algorithmic definition	VUMC^a^		MGB^b^	
PPV^c^	Genotyped, n	PPV	Genotyped, n
*ICD*-or-NLP^d^^, e, f^	0.72	1057	0.58	1379
*ICD*-defined^g^	0.73	640	0.64	725
NLP-only^h^	0.71	417	0.51	654
*ICD*-and-NLP^i^	0.84	515	0.91	335

a VUMC: Vanderbilt University Medical Center.

b MGB: Mass General Brigham.

c PPV: positive predictive values.

d *ICD*: *International Statistical Classification of Diseases*.

e NLP: natural language processing.

f Uses both arms and takes the union of the cases identified by either arm.

g Charts selected based on *ICD*-coded information regardless of whether NLP was positive.

h Charts that lacked *ICD*-coded information but were algorithm positive based on NLP applied to unstructured notes.

i Uses both arms and takes their intersection.

Table 1 summarizes chart review validation results. The *ICD*-and-NLP definition (intersection) achieved the highest PPV of 0.84 at VUMC and 0.91 at MGB, but identified fewer cases (515 and 335, respectively). The *ICD*-or-NLP definition (union) yielded more cases (1057 at VUMC; 1379 at MGB) but lower PPVs (0.72 and 0.58), with 38 of 90 MGB subjects misclassified as OCD cases. These findings highlight the tradeoff between PPV and the number of identified OCD cases when comparing the disjunction (*ICD*-or-NLP) and conjunction (*ICD*-and-NLP) of the 2 arms in the algorithm. The NLP-only definition yielded the lowest PPVs at both sites. Replication at MGB resulted in lower PPVs for 3 of the 4 definitions (*ICD*-defined, NLP-only, and *ICD*-or-NLP), particularly the latter 2, underscoring the challenges associated with using clinical notes across different institutions.

### Description of Algorithm Results

Among 213,275 BioVU participants, our algorithm identified 640 genotyped OCD cases by *ICD* codes (*ICD*-defined), 417 by NLP (NLP-only), 1057 by either arm (*ICD*-or-NLP), and 515 by both (*ICD*-and-NLP). For MGBB, 3385 of 97,275 participants were excluded based on predefined criteria (Figure 1A). Of 93,890 remaining, 53,853 underwent genotyping, yielding 725 *ICD*-defined, 654 NLP-only, 1379 *ICD*-or-NLP, and 335 *ICD*-and-NLP cases (Figure S3 in Multimedia Appendix 1). Pie charts summarizing the OCD case counts are presented in Figure S3 in Multimedia Appendix 1.

Table 2 and Table S2 in Multimedia Appendix 1 summarize demographic characteristics. *ICD*-defined cohorts included older participants (mean age 46.2, SD 17.53 at VUMC; mean 55.4, SD 17.58 at MGB) and a higher proportion with public insurance (55.2% and 65.4%). In contrast, NLP-only cohorts were younger (44.0 at VUMC; 45.2 at MGB) with fewer on public insurance (45.6% and 50.8%). Females predominated across all cohorts, especially NLP-only (65.7% at VUMC; 67.3% at MGB). Most individuals self-reported as White (91%‐92%; at VUMC; 83%‐91%; at MGB), highest in *ICD*-and-NLP cohorts. In terms of hospital utilization, NLP-only cohorts consistently had the fewest visits, diagnoses (*ICD*), procedures (current procedural terminology [CPT]), prescriptions (RxNorm), lab tests (Logical Observation Identifiers Names and Codes [LOINC]), and clinical notes across both sites. The cohort with the highest use varied, being *ICD*-and-NLP at VUMC and *ICD*-only at MGB. Notably, despite lower overall use and a shorter study time frame (1989‐2021 at VUMC vs 1976‐2021 at MGB), VUMC cohorts had markedly more clinical notes, suggesting site-level differences in documentation practices.

**Table 2.** Demographic composition of the Obsessive-compulsive disorder (OCD) cases identified by each arm of the algorithm.

Setting	ICD-defined^a^ (VUMC^c^)	NLP-only^b^ (VUMC)	ICD-defined (MGB^d^)	NLP-only (MGB)
Genotyped, n	640	417	725	654
Age, mean (SD)	46.22 (17.53)	43.99 (15.61)	55.35 (17.58)	45.17 (11.03)
EHR-reported^e^ sex^f^, n (%)				
Female	357 (55.78)	274 (65.71)	442 (60.97)	440 (67.28)
Male	283 (44.22)	143 (34.29)	283 (39.03)	214 (32.72)
Self-reported race, n (%)				
Asian	9 (1.41)	4 (0.96)	10 (1.38)	17 (2.60)
Black or African American	36 (5.63)	21 (5.04)	29 (4.0)	33 (5.05)
White	583 (91.10)	382 (91.61)	646 (89.10)	541 (82.72)
Other	3 (0.47)	1 (0.24)	30 (4.14)	54 (8.26)
Unknown	9 (1.41)	9 (2.16)	10 (1.38)	9 (1.38)
Ethnicity, n (%)				
Hispanic	11 (1.72)	12 (2.88)	12 (1.66)	20 (3.06)
Non-Hispanic	629 (98.28)	405 (97.12)	713 (98.34)	634 (96.94)
Public payer, n (%)				
Yes	353 (55.16)	190 (45.56)	474 (65.38)	332 (50.76)
No	287 (44.84)	227 (54.44)	251 (34.62)	322 (49.24)
Hospital usage, median (IQR)				
EHR length (in years)	7.78 (11.98)	8.53 (12.26)	18.47 (11.62)	14.50 (13.03)
Visit count	76 (127.25)	63 (105)	366 (463.0)	268.5 (362.75)
*ICD* count	208.5 (408.75)	167 (324)	809 (1271.0)	587.5 (809.0)
CPT^g^ count	215 (339.25)	167 (298)	593 (867.0)	405 (595.25)
RxNorm count	117.5 (158.25)	99 (138.75)	566.0 (948.0)	390.0 (696.75)
LOINC^h^ count	159 (129.5)	139.5 (127)	525.5 (532.75)	442.0 (457.75)
Note count	1155 (2255)	1081 (2146.75)	363 (544.0)	273.5 (405.25)

a *ICD*: *International Statistical Classification of Diseases*.

b NLP: natural language processing.

c VUMC: Vanderbilt University Medical Center.

d MGB: Mass General Brigham.

e EHR: electronic health record.

f EHR-reported sex, defined as sex at birth at MGB and self-reported or clinician-recorded sex at VUMC.

g CPT: current procedural terminology.

h LOINC: Logical Observation Identifiers Names and Codes.

### Genetic Analysis

#### VUMC

In the VUMC BioVU cohort, 676 OCD cases (*ICD*-or-NLP) and 46,677 controls passed genotyping quality control. Genetic analyses were restricted to individuals of European ancestry. After applying case-control matching as described in the Methods, the analytic dataset included 398 *ICD*-defined, 278 NLP-only, 676 *ICD*-or-NLP, and 326 *ICD*-and-NLP cases, each at a 1:3 case-control ratio. OCD, MDD, and height PGSs were computed for each algorithm-defined OCD case-control set using summary statistics from previously published GWASs of OCD [14], MDD [28], and height [27]. As shown in Table 3, the OCD PGS was significantly associated with algorithm-defined OCD status in logistic regression analyses for the definitions that incorporated *ICD* codes: *ICD*-defined (odds ratio [OR] 1.22, SE 0.062; *P*=1.51×10^–3^), *ICD*-or-NLP (OR 1.15, SE 0.047; *P*=4.05×10^–3^), and *ICD*-and-NLP (OR 1.26, SE 0.068; *P*=8.19×10^–4^). No significant association was found for the NLP-only definition (OR 1.10, SE 0.072; *P*=.20).

**Table 3.** Association between obsessive-compulsive disorder (OCD) polygenic scores (PGS) and predicted OCD status in Vanderbilt University Medical Center (VUMC) Vanderbilt University Medical Center’s biobank.(BioVU).

Algorithmic definition	Dataset	Number of cases, n	Number of controls, n	OR^a^	BETA^b^	SE	*P* value	*R* ^2^
*ICD*-defined^c^	BioVU^d^	398	1194	1.22	0.198	0.062	1.51E-03	0.88%
NLP-only^e^	BioVU	278	834	1.10	0.094	0.072	.1946	0.21%
*ICD*-or-NLP	BioVU	676	2028	1.15	0.135	0.047	4.05E-03	0.43%
*ICD*-and-NLP	BioVU	326	978	1.26	0.229	0.068	8.19E-04	1.21%

a OR: odds ratio.

b BETA: beta coefficient.

c *ICD*: *International Statistical Classification of Disease*.

d BioVU: Vanderbilt University Medical Center’s biobank.

e NLP: natural language processing.

As shown in Table S7 in Multimedia Appendix 2, the MDD PGS was also associated with algorithm-defined OCD status for the definitions incorporating *ICD* codes, with effect estimates broadly similar to those observed for the OCD PGS. Compared with the corresponding OCD PGS associations, the MDD PGS showed a slightly larger effect estimate and smaller *P* value for *ICD*-defined (OR 1.23, SE 0.061; *P*=6.47×10^–4^), a slightly smaller effect estimate and larger *P* value for the *ICD*-or-NLP definition (OR 1.13, SE 0.048; *P*=1.10×10^–2^), and a similar effect estimate and *P* value for the *ICD*-and-NLP definition (OR 1.26, SE 0.069; *P*=7.78×10^–4^). Full VUMC results for the OCD, MDD, and height PGS analyses, before and after adjustment for MDD status, are provided in Table S7 in Multimedia Appendix 2.

We next assessed whether the associations between PGSs and algorithm-defined OCD status were robust to comorbid MDD by including EHR-derived MDD status as an additional covariate. After adjustment for MDD status, the OCD PGS associations remained directionally consistent across the *ICD*-defined, *ICD*-or-NLP, and *ICD*-and-NLP definitions. These associations remained statistically significant for the *ICD*-or-NLP definition (OR 1.11, SE 0.051; *P*=4.51×10^–2^) and the *ICD*-and-NLP definition (OR 1.20, SE 0.077; *P*=2.13×10^–2^), and were marginal for *ICD*-defined (OR 1.14, SE 0.071; *P*=6.08×10^–2^). In contrast, the MDD PGS associations were attenuated and were no longer statistically significant after adjustment for MDD status: *ICD*-defined (OR 1.10, SE 0.069; *P*=.15), *ICD*-or-NLP (OR 1.04, SE 0.037; *P*=.480), and *ICD*-and-NLP (OR 1.14, SE 0.076; *P*=.08).

To further assess specificity, we examined associations between the height PGS and algorithm-defined OCD case-control status as a negative control analysis. As expected, the height PGS was not significantly associated with any algorithm-defined OCD phenotype, either before or after adjustment for MDD status (*P*>.40 across all analyses).

#### MGB

At MGB, 1379 OCD cases (*ICD*-or-NLP) and 52,474 controls were genotyped across 13 batches on 4 Illumina genotyping platforms. After quality control, duplicated or related samples, non-European samples, and samples and SNPs with low genotyping quality were removed. This left 658 cases and 23,314 controls with 593,590 SNPs in the MEG dataset, and 388 cases and 13,458 controls with 407,608 SNPs in the GSA dataset. After matching controls to cases at a 4:1 ratio based on sex, genotyping batch, age, and genetic proximity, imputation was performed separately for MEG (658 cases, 2632 controls) and GSA (388 cases, 1552 controls). A total of 5,866,159 SNPs passed postimputation quality control in both datasets.

PGSs were computed using GWAS summary statistics of OCD, MDD, and height. As shown in Table 4, logistic regression adjusting for population stratification showed significant associations between OCD PGS and algorithm-defined OCD status for *ICD*-defined (META: OR 1.30, SE 0.048; *P*=7.0×10^–8^), *ICD*-or-NLP (META: OR 1.19, SE 0.035; *P*=5.71×10^–7^), and *ICD*-and-NLP (META: OR 1.32, SE 0.073; *P*=1.53×10^–4^). Conversely, no significant association was found for the NLP-only definition in the meta-analysis (OR 1.08, SE 0.052; *P*=.13), consistent with its lower PPV at MGB (Table 1).

**Table 4.** Association between obsessive-compulsive disorder (OCD) polygenic scores (PGS) and predicted OCD status in MGB Biobank (MGBB).

Algorithmic definition and dataset		Number of cases, n	Number of controls, N	OR^a^	BETA^b^	SE	*P* value	*R*^2^, %
*ICD*-defined^c^								
GSA^d^		185	740	1.47	0.383	0.086	8.86E-06	3.47
MEG^e^		379	1516	1.23	0.204	0.058	4.54E-04	1.02
All		564	2256	1.30	0.260	0.048	7.0E-08	—^f^
NLP-only^g^								
GSA		203	812	1.18	0.166	0.080	3.86E-02	0.66
MEG		279	1116	1.02	0.015	0.068	.8199	0.01
All		482	1928	1.08	0.078	0.052	.1315	—
*ICD*-or-NLP								
GSA		389	1556	1.31	0.267	0.058	4.21E-06	1.74
MEG		661	2644	1.13	0.122	0.044	5.18E-03	0.37
All		1050	4200	1.19	0.174	0.035	5.71E-07	—
*ICD*-and-NLP								
GSA		85	340	1.42	0.351	0.128	6.10E-03	2.83
MEG		164	656	1.27	0.242	0.089	6.91E-02	1.37
All		249	996	1.32	0.278	0.073	1.53E-04	—

a OR: odds ratio.

b BETA: beta coefficient.

c *ICD*: International Statistical Classification of Disease

d GSA: Global Screening Array.

e MEG: Multi-Ethnic Global.

f Not available.

g NLP: natural language processing.

As shown in Table S8 in Multimedia Appendix 2, associations between MDD PGS and OCD status were also observed across the meta-analyzed datasets. In the *ICD*-defined cohort, the MDD PGS showed a slightly smaller effect estimate than the OCD PGS (META: OR 1.21, SE 0.048; *P*=5.81×10^–5^). In contrast, for NLP-only (META: OR 1.21, SE 0.052; *P*=1.93×10^–4^), *ICD*-or-NLP (META: OR 1.21, SE 0.035; *P*=3.40×10^–8^), and *ICD*-and-NLP (META: OR 1.35, SE 0.074; *P*=4.60×10^–5^), the MDD PGS demonstrated larger effect estimates and smaller *P* values than the corresponding OCD PGS associations.

As in the VUMC analyses, we conducted sensitivity analyses including MDD status as an additional covariate. In these adjusted models, MDD PGS associations remained statistically significant but were attenuated across algorithmic definitions. For example, in the *ICD*-and-NLP meta-analysis, the MDD PGS OR decreased from 1.35 before MDD adjustment to 1.29 after adjustment (Table S8 in Multimedia Appendix 2). In contrast, OCD PGS associations remained statistically significant and showed little attenuation after adjustment for MDD status: *ICD*-defined (META: OR 1.29, SE 0.049; *P*=2.45×10^–7^), *ICD*-or-NLP (META: OR 1.17, SE 0.036; *P*=7.88×10^–6^), and *ICD*-and-NLP (META: OR 1.33, SE 0.075; *P*=1.75×10^–4^) definitions. Together with the VUMC results, these findings suggest that OCD PGS associations were more robust to adjustment for MDD status than MDD PGS associations, supporting the relative specificity of OCD-related genetic liability in the algorithm-defined OCD phenotypes.

Finally, consistent with the VUMC negative-control results, no significant associations were found between the height PGS and algorithm-defined OCD status across any phenotype definition, either before or after adjustment for MDD status (all meta-analysis *P*>.20).

## Discussion

### Principal Findings

OCD is a common psychiatric condition, yet remission is achieved in only one-fifth of cases [3]. The average interval between symptom onset and treatment initiation is 7 years [33], and longer duration of untreated OCD is associated with poorer outcomes [34]. Given this, identifying biological markers and genetic risk factors is crucial for facilitating earlier diagnosis and targeted interventions. Large-scale agnostic gene discovery studies of OCD are gaining traction, and recent work demonstrates that discovery yield increases as sample size grows [14]. Previous OCD GWAS have relied on cases ascertained from various sources, including specialist clinics and advocacy organizations [12,13], or national disease registers from the Nordic countries [15,16]. The availability of large-scale, real-world EHR data has provided an opportunity to develop scalable and cost-efficient tools to identify patients with psychiatric disorders, such as OCD, for genetic studies. A prior study used NLP, supplemented by structured diagnostic fields, to ascertain obsessive-compulsive symptoms (OCS) and OCD in a UK mental health case register among patients with schizophrenia, schizoaffective disorder, or bipolar disorder, with the aim of estimating prevalence and examining clinical associations [35]. However, their study was conducted at a single site within a specialty mental health setting and did not include any genetic analysis. In contrast, our study focused on identifying OCD cases from broader patient populations for genetic research, with validation across 2 US health care systems.

In this study, we developed and validated an EHR-based algorithm for identifying OCD that integrates diagnostic codes, medication records, and NLP-derived information from clinical notes. The algorithm achieved high PPV across multiple chart review rounds at 2 health care systems, VUMC and MGB, located in the southeastern and northeastern United States, respectively. We further evaluated whether algorithm-defined phenotypes captured known OCD genetic liability by testing associations with OCD PGS in individuals of European genetic ancestry from BioVU and MGBB. The significant associations observed provide early support that, within this ancestry-defined subset, the algorithm identifies cases with common variant liability overlapping that of traditionally ascertained OCD samples. As outlined below, 3 key findings from our analyses are particularly noteworthy.

First, chart reviews showed *ICD*-and-NLP achieved the highest PPVs (0.84 at VUMC; 0.91 at MGB), outperforming either arm alone (*ICD*-defined, NLP-only) and their union (*ICD*-or-NLP), particularly at MGB, where the PPVs for the *ICD*-defined and NLP-only definitions were only 0.64 and 0.51, respectively (Table 1). These findings suggest the structured diagnostic codes and NLP-derived evidence from clinical notes complement each other in enhancing the precision of OCD phenotype ascertainment. On the other hand, *ICD*-and-NLP identified fewer cases (515 at VUMC, 335 at MGB) for downstream genetic studies, whereas *ICD*-or-NLP captured more (1057 at VUMC, 1379 at MGB) but with substantially reduced PPVs (0.72 at VUMC, 0.58 at MGB). This underscores that the 2 arms are complementary only when used in conjunction since the union of their identified cases (disjunction) tends to accumulate false positives, resulting in poorer PPVs. Moreover, this pattern of findings highlights the tradeoff between achieving high precision (PPV) and maximizing the number of cases identified when selecting an algorithmic definition. Additionally, we observed PPVs achieved by using only *ICD* codes to select cases at the 2 US-based health care systems (PPV=0.73 and 0.64) were notably lower than those reported in the Nordic studies (0.91‐0.96 in Sweden [15], 0.85‐0.96 in Denmark [16]), suggesting less reliability of *ICD*-only ascertainment in the United States compared to Nordic registers.

Second, among individuals of European genetic ancestry, we observed significant associations between OCD PGS from GWAS and algorithm-defined OCD phenotypes, including *ICD*-defined (OR 1.22; *P*=1.51×10^–3^ at VUMC; OR 1.30; *P*=7.0×10^–8^ at MGB), *ICD*-or-NLP (OR 1.15; *P*=4.05×10^–3^ at VUMC; OR 1.19; *P*=5.71×10^–7^ at MGB), and *ICD*-and-NLP (OR 1.26; *P*=8.19×10^–4^ at VUMC; OR 1.32; *P*=1.53×10^–4^ at MGB). These findings provide early support that the proposed algorithm identifies phenotypes that capture common variant liability overlapping with traditionally ascertained OCD samples, supporting their potential integration with traditional cohorts to enhance the statistical power of genetic discovery. By contrast, when used in isolation, the NLP arm yielded smaller effect estimates and weaker OCD PGS associations, underscoring limitations in relying solely on rule-based NLP for ascertaining OCD cases from clinical narratives.

The MDD PGS was also associated with algorithm-defined OCD status, consistent with the high clinical comorbidity and shared genetic liability between OCD and depression. However, sensitivity analyses adjusting for MDD status showed that OCD PGS associations were more robust to this adjustment than MDD PGS associations, supporting the relative specificity of OCD-related genetic liability in the algorithm-defined OCD phenotypes. In addition, height PGS, included as a negative-control trait, was not associated with algorithm-defined OCD status at either site. These findings support the genetic plausibility and relative specificity of the algorithm-defined OCD phenotypes, while also underscoring that PGS enrichment alone should not be interpreted as definitive evidence of diagnostic specificity.

Comparing OCD PGS effect estimates across definitions, *ICD*-defined showed significant associations, indicating that diagnostic codes alone capture substantial common-variant liability. However, *ICD*-only ascertainment has lower PPV in our US EHR systems than in Nordic registers and does not capture OCD-related evidence documented only in clinical narratives. The comparison between *ICD*-and-NLP and *ICD*-or-NLP is therefore more informative for evaluating the precision-yield tradeoff. Although the stricter *ICD*-and-NLP definition yielded numerically higher ORs than the broader *ICD*-or-NLP definition at both sites, consistent with its higher PPV, confidence intervals overlapped substantially. The choice of definition should therefore be guided by the downstream application: for large-scale genetic discovery studies, where statistical power depends heavily on sample size, the broader *ICD*-or-NLP definition may be preferable despite modestly lower genetic enrichment; for studies prioritizing phenotypic precision, such as treatment outcome research, the stricter *ICD*-and-NLP definition may be more appropriate.

Finally, replication across 2 health care systems provides several recommendations for investigators seeking to apply a similar approach in other EHR systems: (1) because variability in local documentation practices and clinical workflows can affect data completeness and algorithm transferability, implementation at a new site should begin with mapping of local structured and unstructured (clinical notes) data, documentation workflows, and relevant clinical infrastructure. For example, the presence of an OCD-specialty clinic may influence how OCD-related evidence is represented in the EHR. Among patients evaluated in these settings, OCD may be more frequently documented in both structured diagnostic fields and narrative notes; at the same time, these same workflows may also generate *ICD* codes related to referrals or text mentions related to screening questionnaires, intake templates, or differential diagnosis. Such mentions may provide useful evidence for OCD phenotyping in some contexts but may also introduce noise for either *ICD*-only or NLP-only algorithms developed in other health care systems; (2) site-specific validation, including local chart reviewing, is recommended before deployment to estimate algorithm performance and identify common sources of false positives. Standardized chart review protocols and reviewer training materials (Multimedia Appendix 1) can help improve reproducibility across sites, while still allowing reviewers to account for local documentation practices; (3) the NLP component of the algorithm should be reviewed and refined against local samples before deployment, particularly for institution-specific abbreviations and acronym collisions, negation patterns, questionnaire and template formatting, and copy-forward text; and (4) when genetic data are available, PGS analyses for OCD and unrelated negative-control traits, such as height, can complement chart review by providing additional evidence that the EHR-derived phenotype captures psychiatric genetic liability overlapping with traditionally ascertained OCD samples. Together, these recommendations support an approach in which the algorithm’s overall architecture and the validation framework can be implemented across sites, while specific components, particularly the NLP rules and the chart review calibration, should be adapted to each health care system’s EHR structure, documentation practices, and clinical context.

### Limitations and Future Directions

Several limitations should be noted: (1) cross-site validation was complicated by variations in data availability and completeness. Diagnostic codes and corresponding clinical notes are not always accessible at the same time, in part because notes may be sequestered or otherwise unavailable, whereas codes remain accessible. This issue is particularly pronounced for psychiatric notes, which have historically had restricted access due to privacy safeguards. Although the ‘Open Notes’ mandate under the 21st Century Cures Act has improved access, adoption remains inconsistent across institutions. Moreover, since patients often receive care across multiple systems, only partial clinical documentation or diagnostic codes may appear within any single site’s EHR, further complicating algorithm replication; (2) rule-based NLP models, including ours, often fail to fully grasp clinical context and struggle with semantic ambiguity due to their reliance on rigid rules. Our analysis at MGB demonstrated this limitation through examples: the NLP arm of the algorithm misclassified cases due to missed negations (eg, “no” responses in poorly formatted questionnaires), copy-pasted text, and abbreviation misinterpretation (eg, “OCD” for osteochondritis dissecans); (3) PGS analyses were restricted to individuals of European genetic ancestry due to the limited availability of GWAS summary statistics for non-European populations. As a result, our PGS findings should be interpreted as supporting the genetic plausibility of the phenotype within the European-ancestry population, and not as evidence of equivalent genetic validity across other ancestral backgrounds. This limitation is not unique to this study but reflects a broader challenge in psychiatric genetics, where the underrepresentation of non-European populations in GWAS limits the cross-ancestry portability of PGS-based validation; (4) although PGS enrichment in algorithm-identified cases demonstrates elevated common variant liability for OCD, it does not establish diagnostic specificity, given genetic correlations and clinical comorbidity across psychiatric disorders. Our sensitivity analyses partially address this concern by showing that OCD PGS associations were more robust to MDD adjustment than MDD PGS associations, and that an unrelated PGS was not associated with OCD status. Nevertheless, residual confounding by psychiatric comorbidity or other clinical factors cannot be ruled out. Furthermore, although we excluded BioVU samples from the GWAS summary statistics used at VUMC and 23andMe samples at both sites, both the GWAS and our algorithm ultimately rely on clinical diagnostic conventions, and the observed PGS associations may partly reflect shared ascertainment processes rather than fully independent biological validation.

This work within the PsycheMERGE network currently focuses on developing a standardized review protocol by training chart reviewers across multiple health care institutions using simulated psychiatric patient records. This standardization and calibration aim to directly address the reduced generalizability of phenotyping algorithms due to inconsistencies in chart review strategies. Recent advances in large language models (LLMs) offer new opportunities for scalable phenotyping without substantial annotated data. Future work will explore using LLMs to identify OCD-related information in clinical notes, supported by recent findings demonstrating superior diagnostic accuracy of LLMs compared to mental health professionals in identifying OCD cases from clinical vignettes [36]. Furthermore, we will diversify the genetic ancestry representation in our OCD validation analyses by using resources such as the All of Us Research Program [37]. This will support the development of more inclusive and equitable models of OCD genetic risk, addressing the limitations of current European-centric studies.

### Conclusions

We developed and validated an EHR-based phenotyping algorithm for OCD integrating diagnostic codes, medication records, and unstructured clinical notes across 2 health care systems (VUMC and MGB). Combining the *ICD* and NLP arms of this algorithm identified OCD cases with high precision, and among individuals of European genetic ancestry, the identified cases showed significant associations with GWAS-derived OCD PGS at both sites. Sensitivity analyses adjusting for MDD status and negative-control analyses using height PGS further supported the genetic plausibility and relative specificity of the phenotype. This scalable, cost-efficient approach can facilitate large-scale genetic studies and advance understanding of OCD’s complex etiology.

## Supplementary material

10.2196/89213 Multimedia Appendix 1 Supplementary materials

10.2196/89213 Multimedia Appendix 2 Supplementary tables S3-S8

## Abbreviations

BDD: body dysmorphic disorder
BioVU: Vanderbilt University Medical Center’s biobank
CBT: cognitive behavioral therapy
CEU: Utah residents with Northern and Western European ancestry
CPT: current procedural terminology
*DSM-5*: *Diagnostic and Statistical Manual of Mental Disorders, Fifth Edition*
EHR: electronic health record
GSA: Global Screening Array
GWAS: genome-wide association studies
*ICD*: *International Statistical Classification of Diseases*
*ICD-10*: *International Statistical Classification of Diseases, 10th Revision*
*ICD-9*: *International Classification of Diseases, 9th Revision*
IRR: interrater reliability
LLM: large language models
LOINC: Logical Observation Identifiers Names and Codes
MDD: major depressive disorder
MEG: Multi-Ethnic Global
MEGA: Multi-Ethnic Genotyping Array
MEGAEX: Expanded Multi-Ethnic Genotyping Array
MGB: Mass General Brigham
MGBB: The Mass General Brigham Biobank
NLP: natural language processing
OC: obsessive-compulsive
OCD: obsessive-compulsive disorder
OCS: obsessive-compulsive symptoms
OR: odds ratio
PGC: Psychiatric Genomics Consortium
PGS: polygenic scores
PPV: positive predictive values
PRS-CS: polygenic risk scores via Bayesian regression with continuous shrinkage priors
PsycheMERGE: Psychiatric electronic Medical Record and Genomics Network
SNP: single-nucleotide polymorphism
VUMC: Vanderbilt University Medical Center
